# Water Source Preferences and Water Quality Perceptions among Women in the Eastern Region, Ghana: A Grounded Theory Study

**DOI:** 10.3390/ijerph16203835

**Published:** 2019-10-11

**Authors:** Jhanel F. Chew, Laura Corlin, Fernando Ona, Sarah Pinto, Esther Fenyi-Baah, Bernard G. Osei, David M. Gute

**Affiliations:** 1 Department of Civil and Environmental Engineering, Tufts University School of Engineering, Medford, MA 02155, USA; Laura.Corlin@tufts.edu (L.C.); David.Gute@tufts.edu (D.M.G.); 2 Section of Preventive Medicine and Epidemiology, Boston University School of Medicine, Boston, MA 02118, USA; 3 Department of Public Health and Community Medicine, Tufts University School of Medicine, Boston, MA 02111, USA; Fernando.Ona@tufts.edu; 4 Department of Anthropology, School of Arts and Sciences, Tufts University, Medford, MA 02155, USA; Sarah.Pinto@tufts.edu; 5 Ghana Ministry of Foreign Affairs and Regional Integration, Accra, Greater Accra, GA-057-0036, Ghana; extabaa28@gmail.com; 6 Farming Systems Ecology, Wageningen University & Research, 6708 Wageningen, The Netherlands; bernardoseei@gmail.com

**Keywords:** Ghana, improved water source, unimproved water source, water preferences, water management, rural water, ethnography, multiple household water sources and uses, seasonality

## Abstract

Residents in the Eastern Region, Ghana with access to improved water sources (e.g., boreholes and covered wells) often choose to collect water from unimproved sources (e.g., rivers and uncovered wells). To assess why, we conducted two field studies to coincide with Ghana’s rainy and dry seasons. During the rainy season, we conducted semi-structured in-depth interviews among a convenience sample of 26 women in four rural communities (including one woman in the dry season). We asked each participant about their attitudes and perceptions of water sources. During the dry season, we observed four women for ≤4 days each to provide context for water collection and water source choice. We used a grounded theory approach considering the multiple household water sources and uses approach to identify three themes informing water source choice: collection of and access to water, water quality perception, and the dynamic interaction of these. Women selected water sources based on multiple factors, including season, accessibility, religious/spiritual messaging, community messaging (e.g., health risks), and ease-of-use (e.g., physical burden). Gender and power dynamics created structural barriers that affected the use of unimproved water sources. A larger role for women in water management and supply decision-making could advance population health goals.

## 1. Introduction

Globally, 663 million people lack access to an improved water source (e.g., piped water or borehole, as defined by the World Health Organization) and eight out of 10 people who lack access to an improved water source live in rural areas [1]. Residents of Ghana, particularly in rural areas, often suffer from inadequate access to improved water sources. The Community Water and Sanitation Agency (CWSA) was established in 1998 to increase access to improved water sources in rural Ghana. Prior to the establishment of this agency, only 28% of the rural population in Ghana had access to improved water sources; this number is now at 70% [2]. In rural settings, multiple household water source use is nearly universal and necessary to provide adequate water for daily use [3,4]. Thus, despite the reported increase in access to improved water sources in many rural regions of Ghana, the use of unimproved water sources is still common [5,6].

Household water source choices in rural Ghana are typically made by women as women are responsible for 64% of the household water collection [7]. Their ability to choose improved water sources over unimproved water sources for household activities depends on water management decisions made at the district level, local level of civil government, and the local level of traditional government. Typically, District Assemblies deal with local government and resource management; however, water resource planning cannot be implemented without input from the Chief of the community [8]. At each level, men have traditionally determined water management policy, despite the dominant role of women in water collection. In recent years, more efforts have been made to include women in formal decision-making processes—for example, by requiring 30% female membership on Water and Sanitation (WATSAN) committees that were established in rural communities as required by the CWSA [9]. Nevertheless, women are still less likely to hold formal positions of power, be involved in the siting of boreholes and wells, or participate in WATSAN committees [9,10]. At the local level, traditional Chiefs (typically male) also influence women’s decision making about water sources and water collection methods. For example, Chiefs enforce cultural norms about which water sources should be used on which days [8,11]. Despite the fact that men hold more formal power regarding water source decision making and women make more of the daily water collection decisions, only one previous study investigated the role of gender in water collection practices [12]. This rural Southern Indian study found that a lack of support from male family members, particularly when a payment for water was required, was a barrier to selecting such a water source, even when female members saw the benefits of collecting the safer water.

Beyond the political and social factors that affect water source choices, it is known that water source preferences are also affected by practical considerations, such as distance to the water source and perceived water quality [5,12,13,14,15]. For example, Kulinkina et al. (2016) found that some residents of rural Ghana chose not to use piped water systems because it tasted salty and it did not create lather when used for washing clothes. Aesthetic characteristics, such as taste, color, and smell also are known to play a role in perception of risk [14,16]. Other studies have noted rationing high quality water for consumptive purposes and matching different source types to different uses of tasks [3,4].

Nevertheless, it is less clear why people would choose to use unimproved water sources over available improved water sources when they perceive the improved water source to be acceptable in terms of taste, smell, and other characteristics. We explored this question by interviewing and observing women in four rural communities in Ghana that were part of the previous study characterizing water quality perceptions to better understand how gender, power, and traditional and civil systems of governance affect water collection, use, and management. [13]. Our objectives for the present study were to (1) assess which water sources women in four rural communities of Ghana used in rainy and dry seasons, (2) understand how seasonality, physical burden, and accessibility affected water source choices, (3) understand how religious/spiritual influences, community influences, and ease-of-use affected perceptions of water quality, and (4) understand how practical considerations and perception of water quality jointly determined water collection practices.

## 2. Materials and Methods

### 2.1. Community Selection

From the 74 communities in the rural agrarian Eastern Region, Ghana previously characterized for water source availability and perception, we selected a sub-sample of four communities for in-depth study [16]. Communities in the sub-sample had water sources that met the water acceptability criteria (*n* = 28; [16]) so that reasons other than aesthetics (e.g., color, scent, taste, absence of visible particles) could be identified for why people continued to use unimproved water sources when the improved water sources that met the acceptability criteria were present. These 28 communities were grouped into four categories: (1) ≥2 functional boreholes (improved water access) and ≥2 perennial river access points (unimproved water access) (*n* = 7); (2) ≥2 functional boreholes and <2 perennial river access points (*n* = 9); (3) <2 functional boreholes and ≥2 perennial river access points (*n* = 6); and 4) <2 functional boreholes and <2 perennial river access points (*n* = 6). Within each category, communities were selected based on population size (eligible communities were between 1000–3500 people) and location. For convenience, all study communities were located within a one-hour drive from Asamankese and were in the West Akim, Upper West Akim, and Ayensuano districts (*n* = 11). One community was selected from category 1 (community A), one community was selected from category 2 (community B), and two communities were selected from category 3 (Communities C and D; see Table A1 for community characteristics). No community was chosen from category 4 because no community met the final selection criteria. Community D was chosen from category 3 due to its physical proximity to Community A. Of the four communities selected, two communities (Communities A and D) collected payment for borehole water only; none of the other water sources in any study community required payment.

### 2.2. Participant Selection

Within each community, participants were recruited via convenience sampling. Women aged 18 years and older were approached at dawn while fetching water or waiting in line at a water source. Verbal consent was obtained for each woman to participate in the study and be audio-recorded. If no women were at the water source, previously interviewed women were asked to recommend women to be interviewed. A total of 25 women were interviewed in May–June 2016 in Phase 1 and one woman was interviewed in February 2017 in Phase 2. One Key Informant was interviewed in each community during the rainy season in Phase 1. For communities A, B, and D, the Key Informant was a current or past member of the community’s Water Committee. In community C, no Water Committee member was available, so a former appointee of the Chief’s Palace was interviewed at the recommendation of the local assemblyman.

For Phase 2, women were selected from among the women who participated in Phase 1. Women were approached at their homes and asked if they would like to participate in the next phase of the study. For community B, no woman who had previously participated in the study consented to be in Phase 2. Thus, a new participant in community B was interviewed in February 2017 and observed the next day.

The Tufts University Social, Behavioral and Educational Institutional Review Board (IRB) classified the May–June 2016 and January–February 2017 fieldwork studies as exempt (Protocols #1605022 and #1612034, respectively)

### 2.3. Phase 1: Rainy Season Observations and Interviews (May–June 2016)

Each participant was observed collecting water and going from their water source to their home. Notes were taken on timing, water collection methods, whether they brought their children, terrain features at the water source, and physical characteristics of the routes taken between the water source and home.

Women also participated in a semi-structured informal interview (*n* = 26). All interviews were audio recorded on an Olympus DS 3500 device (Olympus, Center Valley, PA, USA). All but two interviews were conducted in Twi; the others were conducted in English. A translator trained in health services performed simultaneous translation. The survey questions were adapted from a previously used instrument [5]. The majority of questions were open-ended and addressed participants’ age, time of residence in the community, water collection responsibilities, location of water sources, frequency of water collection activities, and perception of water quality. Not all questions were asked of each participant and the questions were not asked in a specific order. Similar questions were asked of each participant and the order and follow-ups differed based on participant response. All participants were asked whether age and gender affected participation in water collection.

### 2.4. Phase 2: Dry Season Observations (January–February 2017)

We also observed one woman in each community who had previously been interviewed in Phase 1 for up to four days (in Town B, the observation period was one day) (*n* = 4). The four-day period was chosen to capture temporal trends in water collection. Participants were observed conducting normal daily activities in their homes and communities to see if self-reported behaviors during Phase 1 matched observed behaviors in Phase 2.

Casual guided conversations were conducted throughout the observation period to understand (1) the differences between water use in the dry and rainy season, (2) how different water sources were used and perceived, (3) how methods of collection differed by water source, (4) how gender affected the collection, management, storage, and use of water, and (5) perceptions of the effectiveness of water management in the community.

### 2.5. Transcriptions

Participant responses were directly translated from Twi to English during the interview. All interviews conducted in Twi were fully translated and transcribed by two independent translators (including one not involved in the field interviews).

### 2.6. Analysis Methods

A grounded theory approach was used to analyze the interviews [17]. This approach included memo-ing, or writing short notes for each interview (using both sets of transcriptions). It yielded 46 open codes (see Table A2). The open codes reflected groupings of common words, phrases, and concepts used by participants [18]. A second round of coding collapsed the open codes into eight axial codes: (1) good water, (2) bad water (3) methods of collection, (4) outcomes of drinking water, (5) customs around drinking water/spirituality around drinking water (messaging from religious leaders and references to God(s)), (6) comparisons of different water sources, (7) utilization of different water sources for different tasks (matching source to use), and (8) health. Through this process, three major themes emerged: (1) collection of and access to water, (2) perceptions of water quality, and (3) interactions between the first two themes. Results are presented for each of these three major themes. All interviews were coded in NVivo 11.

## 3. Results

### 3.1. Collection of and Access to Water

Fifteen of the 26 women interviewed stated that they regularly use river water (Table 1). Participants were more likely to use river water in Community C, which had at least eight river access points and only one borehole. In contrast, participants were more likely to use borehole water in Community B where there were four boreholes and only one river access point. Well water was also used by four of the 10 participants in Communities B and D.

#### 3.1.1. Seasonality

Participants generally chose water collection methods that were the most convenient and reliable. Therefore, most participants preferred rainwater during the rainy season and borehole or river water during the dry season. In the rainy season, rainwater was viewed as particularly favorable because collection involved minimal effort (open barrels or buckets were placed outside the home). Rainwater was also viewed more positively than river water immediately after rainstorms because storms increased water turbidity.

During the dry season, smaller streams and wells dry up. Participants reported frustration with the unreliability of water access and the length of time needed to collect water at wells during the dry season. Participants stated that they had to pump for one to two minutes to see if water would flow from the spout. Sometimes, they had to change their water collection times to less convenient hours (waking up as early as 4:30 am) or had to wait and come back several hours later. As one participant said, sometimes water collection from the covered well, an improved water source, “doesn’t get finished. You will have to wait before you get water”. A participant discussed the impact this had:

*Water is scarce in this town in the dry season, but we get water in the rainy season. During the dry season, for here, the well can dry up… so it makes us grieve for water here. For the (borehole), it doesn’t dry in the dry season. But all the others dry up. People from other communities come to fetch from the (borehole).*

Water scarcity during the dry season also contributed to an increase in waiting times and the presence of additional non-community members at boreholes (who did not generally contribute to cleaning and maintaining of the boreholes). These issues affected water source decisions for some participants. For example, one participant who lived closer to a borehole than to a river reported that going to a borehole during the dry season was a “waste of time” because of the additional pumping time.

#### 3.1.2. Physical Burden

The physical burden of collecting water is affected both by distance to the water source and by the method of obtaining water from the source. Participants were less likely to travel to farther water sources when closer water sources were available, even if they found the more distal water sources otherwise acceptable. For example, one participant who found both river water and borehole water acceptable generally chose to use river water (“(the borehole) is far from my house and a river lies here. I will not go fetch that far”).

Additionally, more physically demanding water collection methods, such as hand-pumping at boreholes and covered wells, discouraged the use of improved water sources. As one participant said, “after fetching (water from the borehole), you will be hungry. It is difficult to pump and you will get hungry by the time you finish… When my son is on my back and I carry a big (bucket), I will be tired”. This participant reported collecting water from the borehole only once per day and used other water sources as her main sources of water. These issues of physical exhaustion were compounded in the dry season when the water table was low, more people used boreholes, and more physical effort was required to get sufficient quantities of water. One participant said, “you have to pump (the borehole) for a longer time before you get water so you need more energy for the pumping of the borehole… In the dry season, the borehole doesn’t flow well so you would have to stand there for long before you can get water”.

In addition to the physical exhaustion associated with pumping water, pumping can be associated with other negative consequences. This was especially problematic when the pumps were improperly placed or maintained. For example, a participant mentioned that “dress(es) gets wet when you pump (from the well). It leaks when pumping for water and as I stand, you can see my dress is wet”.

#### 3.1.3. Accessibility

Fees, limited hours of operation, waiting times, and cultural factors affected the accessibility of water sources. Boreholes, but not other improved or unimproved water sources, typically had fees associated with water collection. For example, fees were collected for borehole use in two of the four study communities. Fees were collected (primarily by women) for the purpose of cleaning boreholes (sweeping and scrubbing after each use) but the collected fees were not used to repair boreholes. Women reported that borehole water was too expensive for certain water-intensive tasks, such as washing and bathing. These factors created a gender power dynamic that women had little say in how much money water cost and how money was allocated, despite the lived experience of women collecting and using the water for daily tasks. Borehole water also could not be accessed during specific times of the day when boreholes were locked. For example, in two of the four communities, boreholes were only unlocked during peak times (e.g., 5:00–9:00 and 15:00–19:00) since the women who collected the fees had other religious and farming responsibilities. If women needed water during times when the borehole was locked, they chose other water sources.

Cultural practices also affected accessibility. For example, in one town, the Chief said that river water cannot be collected on Thursdays. A participant explained that “our ancestors had that rule, so any Chief that comes has to obey it”. A participant from a different community further explained:

*Everybody has what he or she doesn’t like. So, the river too has its day that it doesn’t want the community to fetch water from it… So, if you go there with your intention, you will die because you are not supposed to go there on Fridays. So if you argue and you go, anything can happen to you.*

On days that the participants could not collect water from certain rivers, they would collect water from other rivers (each river and stream had a different day during which water collection was forbidden) or from other water sources. Generally, women would choose to use water from a borehole if they could not use river water. Women reported that this resulted in long wait times and that they perceived the additional time spent collecting water as wasteful.

### 3.2. Perceptions of Water Quality

When discussing water quality, participants tended to focus on health risks, taste, and aesthetic characteristics. Participants considered water “bad” if it had “germs” (the Twi word for “germs” is the same word as for “small animals”), dirt, leaves, or bodily fluids (e.g., saliva or urine). Participants considered water “good” if it was odorless, cool, tasted good, was “white” (there is no word for clear or colorless in Twi), and lathered well with soap. Perceptions of water quality were influenced by religious and spiritual messages, community messages, and ease-of-use considerations (Table 2).

Overall, the majority of participants (14/26) preferred rainwater to other water sources and all the participants collected rainwater when it was available. This preference was due to both religious/spiritual reasons and practical reasons. From a religious and spiritual standpoint, participants reported sentiments such as “the rainwater is from God so I know that I will not get any sickness when I drink it” and “the borehole is a ground water and the river is God’s creation that flows on the ground as well so the rain water is much cleaner but does not fall always”. Participants also thought that the rainwater was easy to collect and use for household tasks. In contrast, well water (and particularly well water from uncovered wells) was viewed most negatively by participants. Participants were concerned about health risks from well water, stating opinions such as “because it’s from the ground, we can’t say it’s clean…there are particles in it that you can’t see. But as far as the color and the scent, it’s fine. And there’s no cover, so it’s not safe”.

Perception of river water quality was more mixed. Some participants who reported drinking river water instead of water from an available improved water source did so for religious or spiritual reasons. As one participant said, “our great grandparents were using the river… the borehole is new”. The river water was perceived as a more legitimate source of water by some participants. Additionally, many participants reported that the flowing nature of the river made it appear clear, and thus good for drinking and household chores. As one participant stated, “animals fall in it and then leaves too. There might be dirt on the leaves and the water washes it”. For participants who preferred river water, diseases attributed to consumption of river water were considered “common sickness(es) everyone can experience”.

Not all participants felt that the river water was harmless, due primarily to the WATSAN committee messages about the health benefits of borehole water and the risks of water-borne and water-related diseases associated with river water use. Some participants were concerned about diseases including schistosomiasis and guinea worm (guinea worm was recently eradicated in Ghana). As one participant stated, “You will be sick when you take bad water. Like the river. You will see blood in your urine when you swim or bathe. And there are worms like thread which will also go into your body”. While some participants reported boiling or “sieving” river water (letting the dirt settle and then pouring the water into a new container), participants reported that boiling river water adversely affected the taste and at least one participant thought that the boiling process was too cumbersome.

Perceptions of borehole water quality were most strongly and positively affected by community messaging about health. Aesthetic characteristics were used to determine health risks. Water that was considered “clear” was considered “good” water, while “brown” water was considered “dirty” or “bad” water. Five participants mentioned that the borehole water was good because it had been treated (“when they built the borehole, they put some medicine in it and it makes it clean”); none of these participants had seen the borehole being treated or knew if any ongoing treatment occurred, although Water Committee members mentioned treatment of boreholes. This may refer to initial water disinfection when the borehole was built. Participants valued that “no one can step in” or “spit saliva in” the borehole. Nevertheless, ease-of use considerations played a role, as some participants perceived the borehole water as bad quality because it lathered less well with soap, making household tasks more cumbersome.

### 3.3. Interaction of Water Accessibility and Water Quality Perception on Water Source Choice

Accessibility and water quality perception together influenced women’s water source preferences. In situations where easily accessible water was also perceived to be good-quality water, women would choose one primary water source for all household water needs. For example, nine participants preferred river water over borehole water because it was fast and easy to collect, it was perceived to confer low health risks, and the aesthetic characteristics were favorable. For five other participants, borehole water was the preferred water source compared to river water. These participants tended to live closer to boreholes and to perceive more health risks attributed to river water.

Water source choices were more complicated for women who reported a mismatch between accessibility and water quality characteristics. Women typically matched source to use. If a given water source was perceived to be of high quality but was associated with accessibility concerns, women would generally only use that water source for specific functions or at specific times. For example, women who preferred borehole water but either could not always afford the fees or who needed water at times when the borehole was locked might prioritize borehole water for cooking and drinking and use other water sources for cleaning. Similarly, while many participants preferred rainwater over all other water sources when it was available, women would report saving rainwater for either drinking water (among those who thought rainwater tasted best) or washing (among those who thought that rainwater lathered with soap the best).

While women would collect water that was perceived to be of low quality if it was highly accessible (typically characterized in terms of proximity to the water source), this water was usually not used for cooking or drinking. For example, one participant who worked as a cook and whose shop was next to a borehole said that borehole water hurt her stomach, so she only used borehole water for washing pots and pans. Additionally, several women who lived close to wells and thought well water was not as clean as other sources reported using well water for household tasks but not for drinking water.

## 4. Discussion

In our qualitative study, we observed how practical accessibility factors and water quality perceptions jointly affected women’s water source preferences in the Eastern Region, Ghana. Specifically, we considered how water source preferences varied by season, how the physical burden and limited accessibility of some water sources affected water source choices, and how religious/spiritual messages, community messages, and ease-of-use considerations affected water quality perceptions. While women balanced several factors in their daily water source choices, gender and power dynamics also created structural barriers such that women would be less likely to access certain water sources in specific contexts. These previously unexplored questions could lead to efforts to advance gender equity and promote the use of improved water sources.

The practical considerations affecting water choices that we observed are not novel or confined to rural Ghana. Just as we observed that water source preferences varied by season, in peri-urban Cambodia, researchers reported that rainwater is preferred during the rainy season and that aesthetic characteristics affect water source preferences more in the dry season [19]. Similarly, a study conducted in Ghana, Kenya, and Zambia found that improved water sources were used more often in the dry season when other water sources were unavailable [20]. A study in rural Kenya also found that revenue from improved sources increased during the dry season and households were more likely to use unimproved sources when they lived near water sources requiring payment [21]. Not having consistent water sources may decrease hand washing and other basic hygiene practices, thus impacting health outcomes [3].

Practical considerations, such as proximity, accessibility, and ease-of-use, have also previously been found to affect women’s water source preferences [12,13,16,20]. Similarly to other studies, we found that rationing of high quality water (e.g., rainwater) for consumptive purposes such as cooking and drinking, while supplementing with lower quality water (e.g., well water) for non-consumptive tasks, such as laundering and bathing, was common [3,4]. As these practical considerations play a role in matching source to use, it may be worth investigating the potential benefits of reducing the physical burdens of water collection from improved water sources.

As may be expected, we found that both community messaging and religious/spiritual messaging affect women’s water source preferences. This included a narrative that emerged from the oral history of the community as well as messaging from stakeholder groups such as the WATSAN committee. Community messaging most strongly affected women’s perception of the health risks of the water sources. Some women had detailed knowledge of relevant water-borne and water-related illnesses and related these health risks to using unimproved water sources. This was not universally true; however, and some women preferred river water due to religious/spiritual beliefs (although in some locations, religious/spiritual leaders encouraged the use of improved water sources). The influence of religious/spiritual messaging has been seen in other contexts as well; in a study of individuals’ water preferences in Nigeria, strong spiritual ties to water as a “free gift from God” were reported (especially among individuals with less formal education and among community elders) and these beliefs affected water source choices [22]. Therefore, to influence women’s water source choices, it is necessary to consider multiple forms of messaging, including messaging from religious/spiritual leaders.

The interplay between multiple forms of governance, including both traditional and civil forms of governance, structurally affects the management of available water sources [11,23]. Since women in the communities we observed had nuanced knowledge of the strengths and limitations of the available water sources, including women in each of these water governance systems (particularly in a proactive participatory process) could help increase satisfaction, usage, and management of improved water sources [9,23,24,25]. Previous studies found that cultural barriers exist that discourage women from participating in WATSAN committees in Ghana—for example, women are concerned that they will be mocked by other women for their participation and women may be limited from holding certain leadership roles due to illiteracy [10,25,26]. Additionally, time constraints could limit women’s ability to participate in water management. As has been reported in other locations globally, many women in our study reported carrying a double burden of paid and unpaid labor [7,27,28,29]. Successfully including women as decision-makers requires a commitment to examining traditional gender roles, supporting efforts that increase girls’ access to education, and respecting women’s value in improving water management.

### Strengths and Limitations

A major strength of our study was the combined use of in-depth interviews and field observations. We were able to couple detailed information about participants’ reported behaviors and beliefs with their actual behavior. This provided additional context to the participants’ interview responses and increased the validity of the survey results. Another strength of our work was that we conducted field studies in both the rainy and dry seasons. Monitoring efforts are usually heavily biased towards surveys conducted during the dry season [3,4]. By conducting field studies during both seasons, we could comment on seasonal trends in water sources preferences. Our methods allowed for the examination of multiple water source use, which is increasingly seen by policy makers as contributing to achieve improved “household resilience” with regards to water supply and quality [30]. Finally, we examined how gender affects power dynamics related to water management and water collection.

Our study also had several limitations. We were only able to interview a convenience sample of 26 women and four Key Informants in four communities in the Eastern Region. We observed women over a relatively short period of time; it is possible that participants changed their behavior during the observation period to better match what they assumed their observed behavior should be, and these women may have had behaviors or beliefs atypical of their communities. Each of these factors could have limited the generalizability of our findings. Notably, generalizability (transferability) is not the primary objective of the grounded theory methodology of qualitative research. The goal was to build knowledge about multiple household water sources and uses in a novel setting. We suggest that future quantitative studies investigate the water source patterns we observed.

## 5. Conclusions

In the Eastern Region, Ghana, women fulfilled most of the water collection responsibilities. Women’s daily water source choices were based on multiple factors, including seasonality, accessibility, physical burden, spiritual/religious messaging, community messaging, and ease-of-use. Even when acceptable improved water sources were available, women would sometimes choose unimproved water sources when the unimproved water sources were more accessible and when aesthetic characteristics, community messaging, or spiritual/religious messaging suggested that the unimproved water sources did not pose increased health risks over improved water sources. To increase access to and usage of improved water sources, greater inclusion of women in water resource decision-making and management could be helpful.

## Figures and Tables

**Table 1:** Number of participants who use boreholes and river water in each community ^1,2,3,4^.

Community	Borehole Only	River Only	Both	Total
Community A	2	2	3	7
Community B	4	0	0	4
Community C	1	5	2	8
Community D ^1^	3	2	1	6
Total	10	9	6	25

^1^ Depicts water use during the dry and rainy season. ^2^ One woman is excluded from this table because she used only covered well water. ^3^ Three women used well water to supplement borehole and river water. ^4^ All interviewed participants collected rainwater during the rainy season.

**Table 2:** Influence of religious/spiritual messages, community messages, and ease-of-use considerations on perceptions of water quality.

Water Source	Religious/Spiritual Messages	Community Messages	Ease-of-Use Considerations
River water	The river is from God, the river is ancient, and their ancestors drank river water—therefore, river water is good	The river makes people sick because it has germs A schistosomiasis and guinea worm public education campaign discouraged river use River water is clear and flows, therefore it is clean	Sieving or boiling are used, especially for water given to children to drink—but many women do not perceive these steps as necessary Boiling water negatively impacts taste River water lathers well with soap
Borehole water	Ground water is from God, but it can get dirty Religious leaders encourage borehole water use	Community campaigns encourage borehole use to reduce risks of infectious diseases Water committee members say that the water has been treated	Borehole water tastes salty and/or does not taste as good as river water It takes more borehole water to perform household tasks because it does not lather as well with soap
Rainwater	Rainwater is from God and “above” so it is good and healthy		Rainwater tastes good Rainwater easily lathers with soap so less is needed Water is “soft” and makes people feel slippery after bathing Worms grow in the water if stored for too long
Well water	Ground water is from God, but it can get dirty	Well water looks dirty, so it may have diseases	There is insufficient quantity, especially during the dry season

## Appendix A

**Table A1:** Study Community Characteristics.

Community	Population (2014) ^1^	Number of Functional Boreholes	Number of Perennial River Access Points	Number of Covered Wells	Number of Uncovered Wells
Community A	2035	3	2	0	0
Community B	3342	4	1	>2 ^2^	>2 ^2^
Community C	1909	1	>8 ^2^	0	0
Community D	2439	1 (2) ^3^	2	4 (5) ^3^	1

^1^ Based on projections from Ghana Statistical Services, Population and Housing Census, 2010. ^2^ More water sources exist for these locations, as noted by the participants and Key Informants. ^3^ Additional functional sources were noted in January 2017 that were not functional in May 2016.

**Table A2:** Open Codes Grouped into Axial Codes.

Axial Codes	Open Codes
Good Water	The river flows, therefore the water is clean The borehole is clean because it is treated The river protects people Rainwater is soft, lathers well, and/or tastes good The river is perceived as cleaner in the dry season Nothing bad happens when one drinks from the river
Bad Water	The borehole has long wait times when other boreholes break, during the dry season, or on days people cannot use the river River water becomes polluted after mining Rainwater sometimes grows worms The borehole takes a long time to repair when it is broken Well water is unreliable; it often dries up before the rainy season is over The borehole water is “hot”; river water is cooler and/or tastes better River water is not seen as hygienic (anyone can put their foot in the water or spit in the water) The borehole is hard to pump, takes a lot of energy, and may wet the individual pumping
Methods	People usually collect at the water source closest to them Children often share burden of fetching water, especially before and after school Water sources are fairly close to people’s homes People let dirt settle before using the water Boreholes are typically located close together Boiling water is only common when preparing for children People treated the boreholes when they were built or regularly treat the boreholes Women use several methods to keep river water clean (sweep dirt away from the river, women do not bathe, etc.) Rainwater is sometimes treated with camphor to keep out worms River water is “burned” to create a smoky flavor in the water for taste Women use different sized buckets for different tasks
Outcome ^1^	Women buy sachet water for drinking Women complain the borehole tastes salty
Customs around Water/Spirituality Around Water	People do not go to the river on certain days River God/Spirit/Goddess Women will sweep and scrub the borehole when it gets dirty
Use	Women use different types of water sources (e.g., borehole, covered, well, uncovered well, river, rainwater)
Health	Added after interviews anytime someone mentioned disease

^1^ “Outcome” refers to how the water is perceived by participants after collection.
